# Genetic Variation in CNS Myelination and Functional Brain Connectivity in Recombinant Inbred Mice

**DOI:** 10.3390/cells9092119

**Published:** 2020-09-18

**Authors:** Andrea Goudriaan, Maarten Loos, Sabine Spijker, August B. Smit, Mark H. G. Verheijen

**Affiliations:** 1 Department of Molecular and Cellular Neurobiology, Center for Neurogenomics and Cognitive Research, Amsterdam Neuroscience, VU University Amsterdam, 1081 HV Amsterdam, The Netherlands; andreagoudriaan@hotmail.com (A.G.); maarten.loos@sylics.com (M.L.); s.spijker@vu.nl (S.S.); guus.smit@vu.nl (A.B.S.); 2 Sylics (Synaptologics BV), 1008 BA Amsterdam, The Netherlands

**Keywords:** genetics, oligodendrocyte, gene expression, myelin, behavior

## Abstract

Myelination greatly increases the speed of action potential propagation of neurons, thereby enhancing the efficacy of inter-neuronal communication and hence, potentially, optimizing the brain’s signal processing capability. The impact of genetic variation on the extent of axonal myelination and its consequences for brain functioning remain to be determined. Here we investigated this question using a genetic reference panel (GRP) of mouse BXD recombinant inbred (RI) strains, which partly model genetic diversity as observed in human populations, and which show substantial genetic differences in a variety of behaviors, including learning, memory and anxiety. We found coherent differences in the expression of myelin genes in brain tissue of RI strains of the BXD panel, with the largest differences in the hippocampus. The parental C57BL/6J (C57) and DBA/2J (DBA) strains were on opposite ends of the expression spectrum, with C57 showing higher myelin transcript expression compared with DBA. Our experiments showed accompanying differences between C57 and DBA in myelin protein composition, total myelin content, and white matter conduction velocity. Finally, the hippocampal myelin gene expression of the BXD strains correlated significantly with behavioral traits involving anxiety and/or activity. Taken together, our data indicate that genetic variation in myelin gene expression translates to differences observed in myelination, axonal conduction speed, and possibly in anxiety/activity related behaviors.

## 1. Introduction

Axonal myelination increases action potential propagation. In particular, the myelin membrane forces the axonal action potential to be generated only at interruptions of non-myelinated areas of the axon, called the nodes of Ranvier, resulting in a fast, saltatory movement of the action potential along the axon [1,2]. Formation of myelin around neuronal axons is a complex process, which in the central nervous system (CNS) involves oligodendrocytes that provide insulation by locally wrapping their cellular processes around axons. In addition, oligodendrocytes and myelin provide metabolic support to axons, which is crucial for neuron survival and activity [2]. Recent studies have suggested that CNS myelination is driven by two processes: an intrinsic developmental myelination program, and an adaptive myelination process that is stirred by external signals [3,4]. 

In accordance with myelin fundamentally changing the way neural impulses are generated and transmitted, loss of myelin integrity can lead to severe neurological symptoms [1,2]. With the findings that activity-dependent myelination may underlie an additional mechanism of nervous system plasticity [5,6,7,8,9], myelin also attracted interest as contributor to cognitive performance and behavior, including general intelligence via regulation of conduction velocities in the brain [10,11,12] as well as a wide range of psychiatric disorders, such as schizophrenia, autism, mood disorders and drug addiction [7,13,14,15,16,17,18,19,20]. Several inherited myelin disorders have been identified with a Mendelian mode of inheritance. However, predisposition to complex traits and psychiatric diseases is often characterized by genetic heterogeneity and a high probability of complex gene-by-gene and gene-by-environment interactions [21,22]. To what extent variation in the expression of myelin genes contributes to complex behavioral traits and diseases is largely unknown. 

Genetic reference populations (GRPs) in animals, for instance panels of recombinant inbred (RI) strains, have been assembled to model, at least in part, the genetic complexity in human populations, while enabling tight experimental control and allowing extensive replication studies [23,24,25]. The BXD mouse resource is currently one of the largest and best characterized mouse GRPs, composed of ~160 lines that descend from crosses and inbreeding of the parental lines C57BL/6J (C57) and DBA/2J (DBA) [23,26]. A major advantage of BXD RI strains is that experimental expression data and phenotype data for many complex traits are publicly available, allowing for hypothesis generation. Genetic differences between C57 and DBA mice have been shown to translate into a broad spectrum of CNS related functional and molecular correlates, for example, differences in activity, impulsive action, hippocampal related memory and learning tasks, post- and pre-synaptic protein expression, and synaptic transmission and plasticity [27,28,29,30,31,32,33,34,35,36,37,38,39,40]. Through genetic linkage analyses, the genetic and phenotypic differences in the BXD panel of RI strains have resulted in identification of genes and loci involved in complex CNS functions, such as impulsivity [41], reversal learning [42], attention [43], neuronal oscillations [44], hearing loss [45], and fear and spatial learning [39,40]. Possible differences in myelination between BXD RI strains, and the relation with their behavioral differences, have not yet been investigated. In the present study, we set out to investigate variation in myelin gene expression in the BXD GRP using expression data from the publicly available GeneNetwork database (www.genenetwork.org). We observed profound differences in the expression of myelin genes over more than 70 BXD strains, correlating with behavioral phenotypes of activity and/or anxiety. The parental strains C57 and DBA were on opposite ends of the expression spectrum, with C57 showing higher myelin transcript expression. Furthermore, C57 and DBA showed differences in myelin fiber size and white matter conduction velocity. We conclude that the differences in myelin fiber size might contribute to functional and/or behavioral differences in these strains. The BXD GRP might represent a promising genetic resource to further disentangle the molecular myelin substrates that influence variation in brain connectivity, and cognitive and behavioral traits.

## 2. Materials and Methods

### 2.1. Clustering of BXD Gene Expression Data

Gene expression data from BXD mice strains were derived from the GeneNetwork database: http://www.genenetwork.org/webqtl/main.py. The GeneNetwork database provides open access to BXD and other RI strain derived microarray data, single nucleotide polymorphism (SNP) data, and phenotypic data for quantitative trait loci analysis and gene expression correlation analyses.

Gene expression data were exported for manually selected probes in the PDNN hippocampus database (Hippocampus Consortium M430v2), and the PDNN whole brain database (INIA Brain mRNA M430). The Hippocampus database was chosen as one of the most elaborate brain databases, as well as most highly recommended dataset on GeneNetwork itself (http://www.genenetwork.org/webqtl/main.py?FormID=sharinginfo&GN_AccessionId=112). PDNN normalization was chosen, as this takes into account bias due to differential probe hybridization [46]. Analyses were performed including data for all BXD strains, parental C57 and DBA strains, and the two reciprocal F1 hybrids (N of strains is 71, pool size: 2–4 mice per strain). Analyses were repeated with the PDNN whole brain database and were performed including data for BXD strains, and parental C57 and DBA (N of strains is 41, pool size: 3 mice per strain).

Selected probes included those for myelin transcripts, as well as for transcripts involved in lipid metabolism and axonal/dendritic markers. Gene expression data were only included for probes fulfilling the following criteria: (1) 100% gene specificity as confirmed by Basic Local Alignment Search Tool (BLAST), (2) located on exons, and (3) with log2 expression values of >8, to prevent background noise in the data. The BXD expression data were scaled per probe by subtracting per probe, the total median of all BXD strains from the exported mean-value per strain. Thus, showing for each probe the deviation per strain in comparison to the median expression over all strains. If multiple probes per gene were present, the average for those probes was taken. Clustering was performed with TMEV software (http://mev.tm4.org) using Euclidean distance and average linkage clustering.

### 2.2. Animals

C57BL/6J and DBA/2J were obtained from Charles River (Charles River Laboratories, L’Arbresle, France, the European supplier of JAX mice, Jackson laboratories, Bar Harbor, ME, USA), and were housed with cage enrichment and water and food ad libitum on a 12/12 h rhythm lights on/off with lights on at 7 a.m. All experiments were approved by the local animal research committee (Dierexperimentencommissie VU University, protocols: MCN13-01 (2013); MCN14-16 (2014)) and complied in accordance with the European Council Directive (86/609/EEC).

### 2.3. Preparation of Brain Extracts

Brains or hippocampi were freshly dissected on ice from C57 and DBA mice (postnatal day P20, and adult mice aged 8 to 12 weeks), as previously described by us [47]. In short: brains were separated from the spinal cord, medulla oblongata, and cerebellum. When hippocampi were dissected, special care was taken to prevent contamination by the corpus callosum, the rest of the brain was kept and referred to as ‘rest of the brain’. Brain and hippocampi preparations were cleaned from the meninges, rapidly frozen in isopentane and on dry ice, and kept at −80 °C until used. On use, the tissue was homogenized in homogenization buffer consisting of 5 mM HEPES/NaOH, pH 7.4, 0.32 M sucrose, 0.016 U/mL of RNase inhibitor (Invitrogen, Carlsbad, CA, USA), complete EDTA-free, protease inhibitory cocktail (1 tablet/50 mL of homogenization buffer from Roche Applied Sciences, Indianapolis, IN, USA). This homogenate was used for all biochemical studies on tissue of the hippocampus and whole brain tissue.

### 2.4. RNA Isolation, Reverse Transcription, and Quantitative PCR

Total RNA from the hippocampus and rest of the brain from C57 (n = 6) and DBA (n = 5) mice, aged 10 to 12 weeks, was isolated using TRIzol (Invitrogen), according to the manufacturer’s protocol. Synthesis of cDNA and quantitative PCR (qPCR) reactions were performed as described previously [48]. The primer sequences are shown in Supplementary Table S1. Results were normalized using a selection of housekeeping genes according to Vandesompele et al. [49].

### 2.5. Immunoblotting on Brain Extracts

Whole brain homogenates (including the hippocampus) were mixed with SDS sample buffer and heated to 90 °C for 5 min. Proteins were separated by SDS-PAGE in a Mini-Protean electrophoresis system (Bio-Rad Laboratories, Hercules, CA, USA) and electroblotted overnight onto polyvinylidene difluoride membranes. Membranes were probed with primary antibodies against Myelin-associated glycoprotein (MAG) (Mouse anti-mouse/rat/human, 1:1000, Abcam), Myelin-associated oligodendrocyte basic protein (MOBP) (Rabbit anti-mouse/human, 1:500, Abcam), 2′,3′-Cyclic nucleotide 3′-phosphodiesterase (CNP) (Mouse anti-mouse, 1:2000, Sigma), myelin oligodendrocyte glycoprotein (MOG) (Rabbit anti-mouse, 1:500, Sigma), Myelin basic protein (MBP) (Rat anti-mouse, 1:50, Abcam), Claudin11 (Rabbit anti-mouse/rat/human, 1:100, Santa Cruz). The blots were washed and incubated for 1 h at room temperature (RT) with AP-conjugated secondary antibody (1:1000; Dako, Glostrup, Denmark). Immunodetection was performed using the ECF immunoblotting detection system for AP-conjugated secondary antibody (GE Healthcare, Diegem, Belgium). Blots were scanned with the FLA-5000 (Fuji Photo Film Corp.). Relative amounts of immunoreactivity were quantified using Quantity One 1-D software (Bio-Rad). To correct for input differences, Coomassie protein staining (the upper or lower half of the same gel for ECF) was used.

### 2.6. Conduction Velocity Measurements

Conduction velocity of fibers in acute prepared coronal sections of the corpus callosum was determined as previously described [50] for C57 (n = 4) and DBA (n = 5) mice, at 2 months old. Evoked compound action potentials were measured by extracellular field recordings using two different recording electrodes and were abolished by tetrodotoxine (TTX), a selective blocker of voltage-gated sodium channels.

### 2.7. Electron Microscopy (EM) and Morphometric Analysis

EM analysis of myelin was performed as previously described [50]. Each group consisted of at least 3 mice at P60, and 120–140 axons per mouse line were counted.

### 2.8. Behavioral Measurements and Correlations with Myelin Expression Data from Hippocampus

Behavioral testing was performed by using the Open field assay, 5-choice serial reaction time task (5CSRTT), pre-pulse inhibition test (PPI) and Dark light box, and described by us previously [43,44,51], except for the Dark light box. 

Dark light box: mice were introduced into the dark compartment (<10 lux, length × width × height: 25 cm × 25 cm × 30 cm) of a dark light box. Then, 60 s later a door opened providing access to an identical sized compartment that was brightly lit (325 lux) and left open for 10 min. Visits to, and time spent in, the light compartment were counted when the body reference point of a mouse protruded at least 2 cm into the light compartment away from the door, as assessed by video tracking (Ethovision 3.1, Noldus Information Technology, Wageningen, The Netherlands).

Analyses were performed on BXD strains (n = 33 to n = 40, depending on the test), with n > 10 individuals per strain. Pearson correlations between the mean myelin gene expression value per strain as derived from Hippocampus Consortium M430v2 data set, and the mean behavioral data per strain, were performed on either raw data, or Log10 transformed data when normality was not present.

### 2.9. Statistical Analysis

Statistical differences between C57 and DBA for data using Q-PCR, electron microscopy, immunoblotting and conduction velocity were analyzed using Student’s *t*-test, unless otherwise indicated in the legends. Statistical numeric data are provided in the text (Results and legends). Data are presented as mean ± SEM. 

## 3. Results

### 3.1. Myelin Gene Expression Shows Large Differences between C57, DBA and Their BXD Progeny

**Figure 1:**
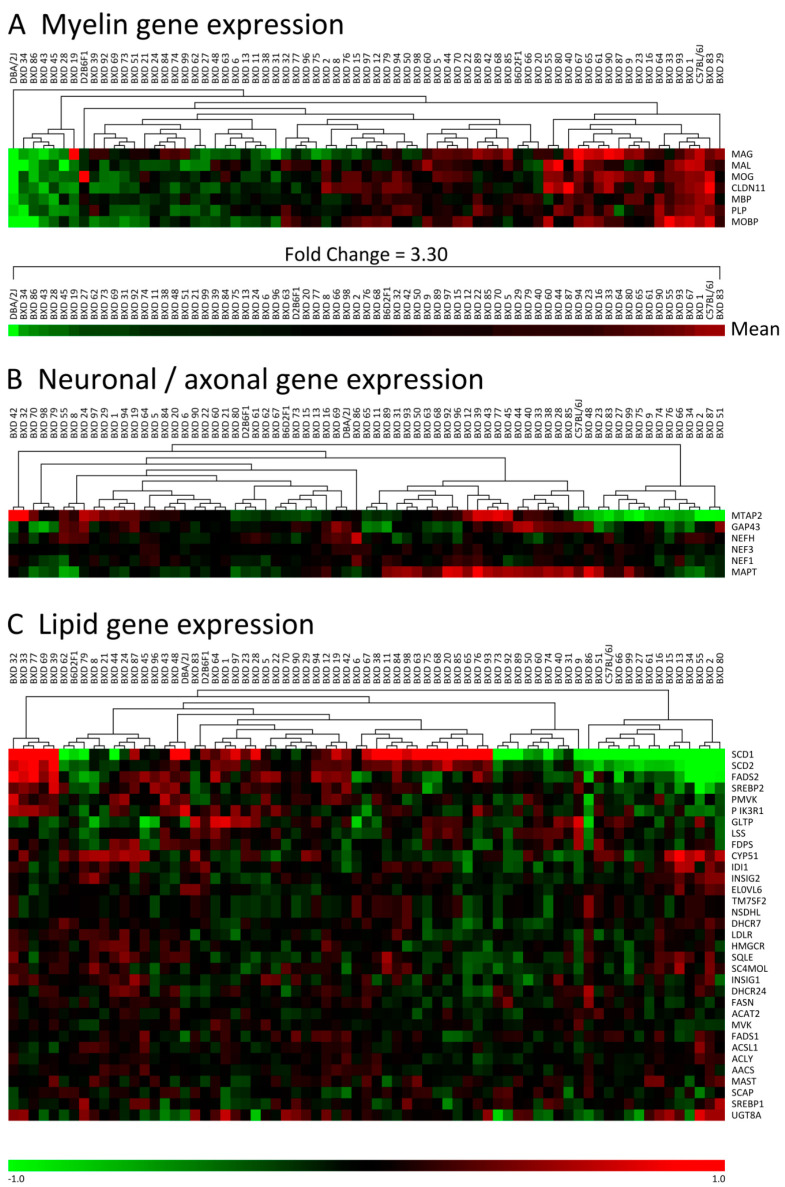
Clustering of gene expression data shows pronounced differences in myelin gene expression between C57BL/6J (C57), DBA/2J (DBA), and their BXD progeny. BXD microarray data were obtained from the GeneNetwork ‘Hippocampus Consortium PDNN’ dataset. Transcripts were selected and their gene expression patterns clustered using Euclidean distance and average linkage clustering. (**A**) The clustering pattern indicates a clear group regulation of all myelin transcripts investigated, with the parental strains C57 and DBA opposite at the most extreme ends. (**B**,**C**) Clustering of transcript data related to axonal/dendritic markers and lipid metabolism, respectively, did not reveal similar patterns of co-regulation. The number of strains is 71. The scale bar is depicting Log2 gene expression values. The intensity threshold was set to 0 for all depicted functional groups (i.e., myelin, axonal/dendritic and lipid genes).

**Figure 2:**
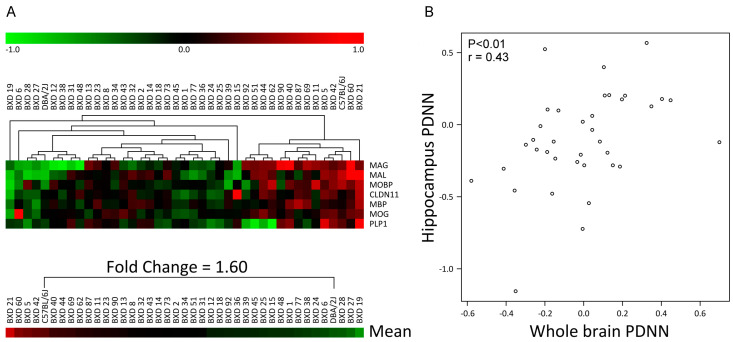
Clustering of gene expression from the whole brain INIA Brain dataset shows pronounced differences in myelin gene expression between C57, DBA, and their BXD progeny. BXD microarray data for the whole brain were derived from the GeneNetwork ‘INIA Brain PDNN’ dataset. (**A**) Myelin transcripts were selected, and their gene expression patterns clustered using Euclidean distance and average linkage clustering. A clear clustering pattern involving all myelin transcripts was detected, with the parental strains C57 and DBA at the most extreme ends. The number of strains is 41. The scale bar is depicting Log2 gene expression values. The intensity threshold was set to 0. (**B**) Correlation between the INIA Brain PDNN and the independent Hippocampus Consortium PDNN database. Pearson correlation r = 0.43, *p* < 0.01. The number of strains is 37.

To determine possible myelin gene expression differences between C57, DBA and their BXD offspring, expression data were extracted from the GeneNetwork database using the high quality brain dataset Hippocampus Consortium [46]. Various transcripts were investigated that are known to be associated with different myelin compartments, e.g., compact myelin (myelin proteolipid protein (PLP), MBP, MAL, MOBP), non-compact myelin (MAG, MOG), and radial component (Claudin11). Cluster analysis of the myelin transcripts identified strong differences between BXD strains, with a pattern that was very consistent over all the myelin genes selected, i.e., if a BXD strain showed a higher or lower expression of a myelin gene compared with the median of this gene for all BXD strains combined, this would in general be seen for all other myelin genes as well (Figure 1A). Importantly, this co-regulation pattern of genes over all BXD strains was not found for other functional groups, i.e., gene expression of neuronal/axonal markers (Figure 1B) and lipid metabolism (Figure 1C). The mean difference in the expression of the myelin gene group between strains was a maximal 3.3-fold. Both the parental strains, C57 and DBA, were on the opposite extreme ends of the myelin gene expression spectrum. This analysis was successfully replicated for whole brain, in the smaller INIA Brain mRNA database (Figure 2).

### 3.2. qPCR Analysis Independently Confirms Significant Differences in Myelin Gene Expression between C57 and DBA Mice

**Figure 3:**
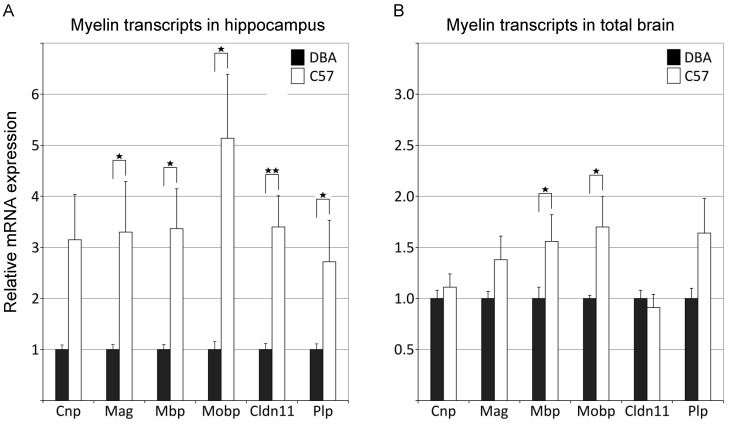
qPCR analysis confirms myelin gene expression differences between the parental C57 and DBA strains. (**A**) qPCR was performed on myelin gene transcripts in hippocampi from C57 and DBA mice. For nearly all myelin genes investigated, gene expression differences were significant, with C57 mice showing higher levels of expression. (**B**) qPCR analysis on myelin gene transcript in total brain tissue (minus hippocampus). DBA, n = 5; C57, n = 6. *t*-test (1-tailed) ** *p* < 0.01; * *p* < 0.05.

To validate the microarray-based data from GeneNetwork, qPCR analysis was performed both on hippocampi and on tissue from the rest of the brain derived from adult parental strains C57 and DBA. The hippocampi of C57 mice showed three to five-fold higher levels of myelin transcript levels than DBA (Figure 3A). Similar higher levels for myelin transcripts were found in total brain (brain minus hippocampus), albeit to a lesser extent (Figure 3B). Together these data confirm that C57 and DBA are different in the expression of myelin genes.

### 3.3. Protein Analysis Reveals Differences in Myelin Protein Expression between C57 and DBA Strains

**Figure 4:**
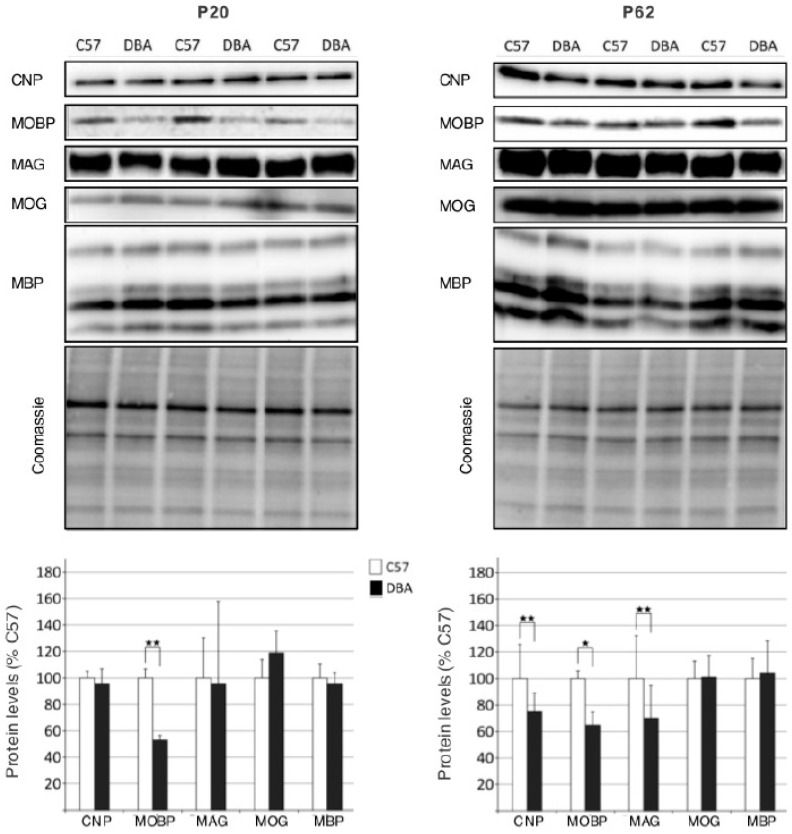
Differences in myelin protein expression between C57 and DBA mice in whole brain extracts. Immunoblotting was performed for selected myelin proteins on whole brain extracts of P20 (**A**) and P62 (adult) mice (**B**). Coomassie staining was used for normalization to control for differences in protein. Graphs show the quantification of myelin proteins measured in pups (P20, left panel) and adult (P62, right panel) mice, respectively. Significant expression differences represented by *t*-test (2-tailed) ** *p* < 0.01; * *p* < 0.05. n = 3–4 mice per group.

To check whether the genetic differences in myelin transcript levels also extended to protein levels, immunoblotting from whole brain homogenates (including hippocampus) was performed on several proteins associated to the different myelin compartments (Figure 4). To determine potential developmental effects, the analysis was performed on P20 (pups) and P62 (adult) mice. Myelin protein MOBP was found to be higher expressed in C57 in both pups and adults, whereas CNP and MAG were higher expressed in C57 adults only. Again, no differences were found for a selection of axonal/neuronal markers (Figure S1). Thus, for a subset of the myelin transcripts, their higher levels in C57 mice were followed by higher myelin protein levels.

### 3.4. Electron Microscopy (EM) Reveals Smaller Myelinated Fibers in DBA Mice

**Figure 5:**
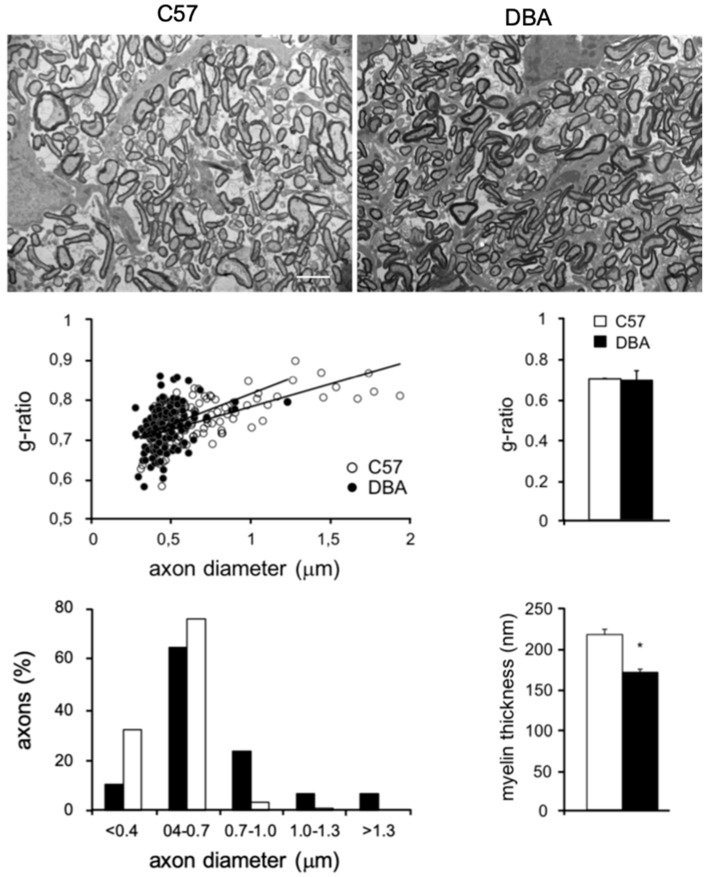
Smaller myelinated fibers in optic nerves of DBA mice. Electron microscopy (EM) analysis of optic nerves in cross-sections of either C57 or DBA, respectively, in mice at 2 months of age. Line graph: morphometric g-ratio analysis of myelinated axons in optic nerves of C57 and DBA. Bar graphs: morphometric analysis of myelinated axons showing g-ratio (averaged), axon diameter and myelin thickness for myelinated fibers. Scale bar, 2 μm. Significant expression differences: *t*-test (2-tailed) * *p* < 0.05.

To further investigate myelin protein differences between C57 and DBA at 2 months of age, EM analysis was performed on optic nerves, a CNS structure in which all axons are myelinated, making it possible to determine small differences in CNS myelination (Figure 5) [1,2,50,52,53,54]. No differences in g-ratio were found between C57 and DBA, whereas the myelin thickness was smaller in DBA compared with C57 (Figure 5). This is in line with the higher levels of myelin transcripts (Figure 1, Figure 2 and Figure 3) observed in C57, and higher levels of a subset of myelin proteins (Figure 4). Based on a similar g-ratio and smaller myelin thickness in DBA, this would imply that DBA mice have smaller diameter axons. We indeed confirmed that myelinated fibers in the optic nerve are smaller in DBA compared with C57 mice (Figure 5).

### 3.5. Higher Axonal Conduction Velocities in Myelinated Axons of C57 Mice

**Figure 6:**
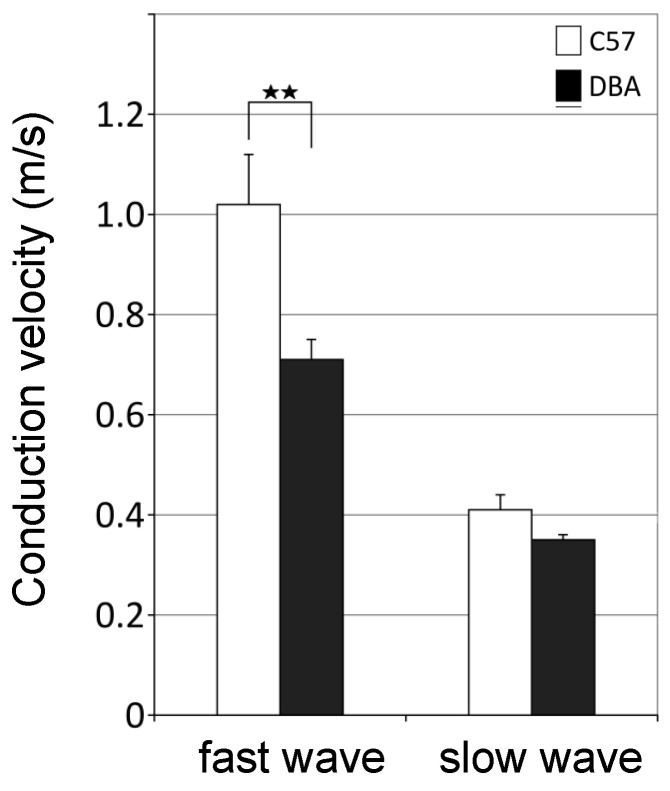
DBA mice show, in comparison with C57, reduced signal conduction velocity for fast, myelinated fibers in the corpus callosum. A significant difference in conduction velocity was observed for the fast wave between DBA and C57, as depicted. See Figure S2 for example trace of slow conducting fibers (slow wave) and fast conduction fibers (fast wave). Significant differences are represented by *t*-test (2-tailed) ** *p* < 0.01. C57, n = 4 (14 slices); DBA, n = 5 (11 slices).

To determine whether the observed differences in myelinated fiber size may underlie differences in the velocity of action potential propagation in the brains of C57 versus DBA mice, we measured conduction velocity in brain slices of C57 (n = 5) and DBA (n = 4) mice at 2 months of age (Figure S2). Since the corpus callosum is the most prominent white matter structure in the rodent brain, it is likely to strongly contribute to the observed differences in whole brain abundance of myelin transcripts and proteins between C57 and DBA. Accordingly, higher conduction velocities were found for the fast conducting fibers in the corpus callosum of C57 mice when compared with DBA mice (Figure 6). Because fast conducting fibers are myelinated, in contrast to the slower conducting fibers, this indicates that the differences observed in myelinated fiber size contribute to differences in functional connectivity in the brains of C57 and DBA mice.

### 3.6. Hippocampal Myelin Gene Expression Correlates with Behavioral Traits in C57, DBA and Their BXD Progeny

**Figure 7:**
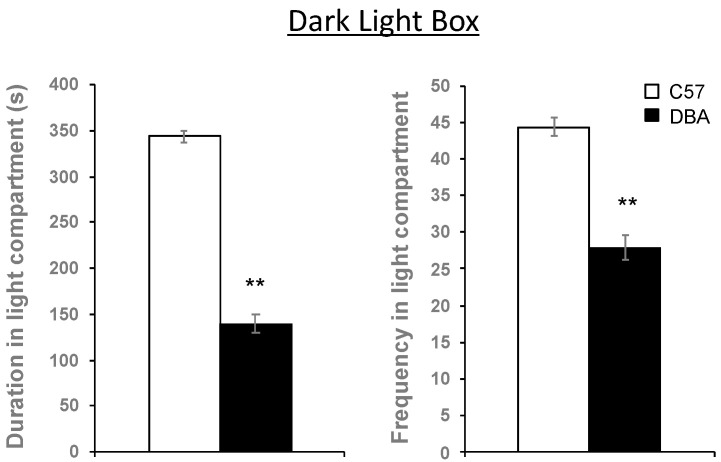
DBA mice show, in comparison with C57, increased anxiety levels in the dark light box. A significant difference in time spent (duration) and in the number of visits (frequency) in the light compartment of the box was observed between DBA and C57, as depicted. Significant differences are represented by *t*-test (2-tailed) ** *p* < 0.01. C57, n = 52; DBA, n = 61.

Next, we investigated whether the differences in myelin gene expression, and white matter conduction velocity might relate to behavioral differences in various domains observed between C57, DBA and their BXD offspring, as we previously assessed [41,43,44], except for the dark light box for which results have not been previously published. Reduced duration and visits (frequency) to the light compartment of the dark light box were found for DBA compared to C57 mice (Figure 7), indicating higher anxiety levels of DBA mice. Significant positive correlations were found between myelin gene expression in the hippocampus and specific behavioral measures of activity and/or anxiety in the open field and the dark light box (Table 1).

This indicates that higher myelin transcript levels might be related to higher activity levels and/or less anxiety as measured by moving around longer and more frequently in open and light spaces and compartments. Correlations were not significant for measures of impulsivity and attention as determined by the 5-choice serial reaction time task (5CSRTT), and for measures of sensorimotor gating in a pre-pulse inhibition test (PPI). Taking these results together, we conclude that the myelin gene expression level correlates with activity and/or anxiety levels of BXD mice, indicative of a role for genetic variance in myelination in brain functioning and behavior. 

## 4. Discussion

The BXD panel of mouse RI strains is a GRP for modeling of genetic complexity, such as that present in the human population [23]. The parental strains, C57 and DBA, and their offspring are known to differ in many behavioral, molecular and functional correlates. Here, we set out to investigate whether genetic differences in myelin transcription are present in BXD lines, which may underlie brain molecular, functional and behavioral differences in these strains. 

We found robust differences in expression of myelin transcripts in a BXD panel, for all myelin genes investigated. These results are in line with the literature on myelin gene expression, indicating the presence of transcription factors that specifically regulate expression of groups of oligodendrocyte and myelin genes [53,55,56,57,58]. The parental C57 and DBA strains were on the opposite ends of the expression spectrum, with higher myelin transcript levels in C57. We found that the differences in transcript levels did translate to higher protein expression in C57 for myelin-associated proteins MOBP, CNP and MAG, the latter in adults only. The fact that not all myelin gene expression differences found on transcript level were reflected at the protein level, is not uncommon considering the many regulatory processes that can occur in-between gene transcription and protein expression [59]. In addition, based on the observation that during the developmental peak of myelination (P20), DBA had only lower myelin protein levels for MOBP, one may speculate that the observed larger differences in adults are rather the result of differences in adaptive myelination than in early developmental myelination [3,4]. 

Importantly, our conduction velocity measurements showed specifically reduced action potential propagation for the fast, myelinated fibers in the corpus callosum of DBA compared with C57, which indicates that the differences in myelin gene expression might underlie functional differences in brain connectivity and signaling. Accordingly, we found that myelinated fibers of DBA had smaller axon diameters and thinner myelin. The diameter of myelinated axons is a well-known feature that influences the speed of signal transduction in axons [54]. Interestingly, smaller caliber myelinated fibers are reported for mutant mice for each of the myelin proteins that we observed which are lower expressed in DBA mice: MAG, MOBP and CNP [52,60,61,62], which is in line with the established concept that proper myelination provides, as well as insulation, also trophic support for axons, thereby increasing axon caliber [2]. Together, our observations on myelination differences between C57 and DBA provide evidence for the genetic underpinning of myelination. This in accordance with recent findings that genetics influence myelination of the human cortex [63]. The observed changes in protein levels for only a subset of myelin proteins may cause subtle changes in myelin ultrastructure and consequently in conduction velocity, which remains to be determined.

**Figure 8:**
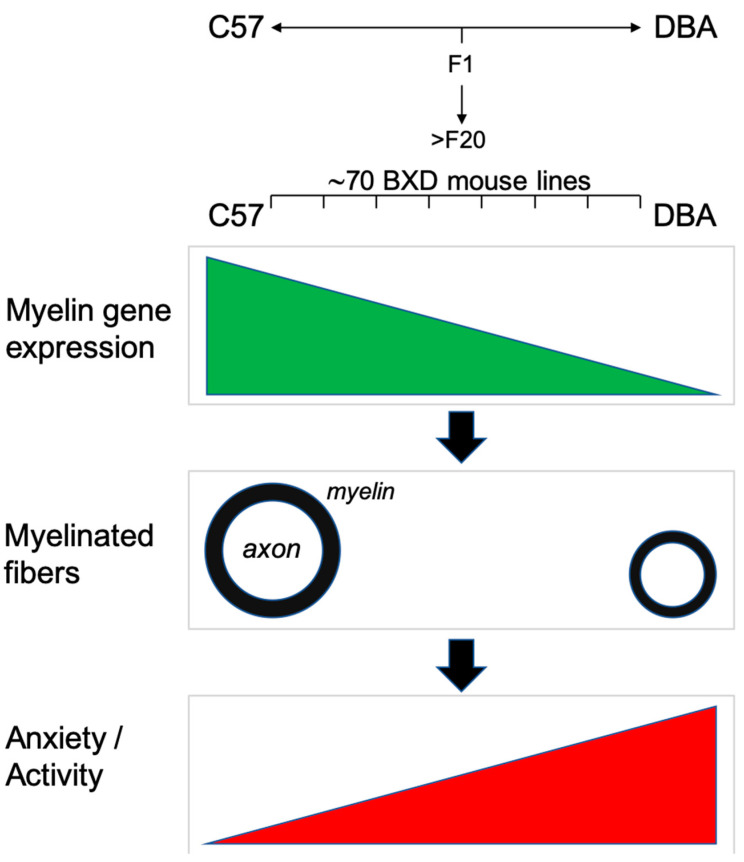
A summarizing view on the correlation of myelin gene expression, myelin fiber diameter and behavioral traits involving anxiety and/or activity.

Interestingly, recent studies point to conduction velocity and processing speed associated with myelinated fibers as critical regulators of cognition in health and disease [7,10,11,12,13,14,15,16,17,18,19,20]. We hypothesized that the observed genetic variation in myelin gene expression, and difference in functional connectivity, might relate to behavioral differences between C57, DBA and their BXD offspring, as we previously assessed [41,43,44]. Accordingly, we found that the BXD myelin gene expression data correlate with differences in anxiety/activity behavioral traits (Figure 8). The correlations were only found for anxiety/activity behavioral traits, and not for measures of impulsivity or pre-pulse inhibition (PPI), indicating that the here reported genetic differences in myelination might influence specific behaviors only. It should be noted that myelin gene expression was determined under non-stimulated (home cage) conditions. With the recent findings that myelination is plastic and can be regulated by external signals (adaptive myelination [3,4,5,6,7,8,9]), it will be interesting to determine whether the genetic difference in myelination between C57, DBA and their offspring is, furthermore, affecting myelin plasticity, e.g., possibly becoming even more prominent when animals undergo behavioral stimulation.

Noteworthily, DBA mice are often selected as an animal model for specific research purposes: e.g., DBA show distal axonal injuries of the optic nerve at an early age [64], and DBA mice are more vulnerable than C57 for kainic acid induced neurodegeneration [65]. Moreover, general differences in synapse and circuitry formation between C57 and DBA are well appreciated and studied [35,44,66,67,68,69]. It remains to be determined whether the differential expression of myelin genes in C57 and DBA may also contribute to these features.

In conclusion, we report significant differences in myelin between C57 and DBA on the molecular level (myelin transcripts and proteins), structural level (size of myelinated fibers) and functional level (conduction velocity). Together with the observed differences in myelin gene expression and behavior of their BXD offspring, we propose that genetic variation in myelin gene expression translates to differences observed in myelination, axonal conduction speed, and possibly in anxiety/activity related behaviors.

## Figures and Tables

**Table 1:** Myelin gene expression of BXD lines correlates with behavioral phenotypes.

Task	Parameter	*p*-Value	r	n
Dark light box	***Duration in light compartment***	*0.040* *	***0.31***	***38***
	***Frequency in light compartment***	*0.024* *	***0.37***	***37***
Open Field	***Total distance travelled***	*0.023* *	***0.37***	***38***
	***Frequency in center***	*0.021* *	***0.38***	***38***
	Duration in center	0.168	0.23	37
5-choice serial	Number of premature pokes	0.455	−0.12	40
Reaction time task	RT-correct	0.092	−0.27	40
	Accuracy	0.695	0.07	39
	Omissions	0.567	0.09	40
Pre-pulse inhibition	PPI65	0.274	0.20	33
(PPI)	PPI70	0.496	0.12	34
	PPI175	0.605	0.09	33

Significant correlations are shown in **bold** and ***cursive***. * *p* < 0.05. Abbreviations: r = Pearson product–moment correlation coefficient, n = number of BXD strains, including parental C57 and DBA. N of mice per strain for myelin gene expression values (from GeneNetwork BXD hippocampus consortium) varies between 2 and 4, for behavioral analysis >10 (as described in Methods).

## Supplementary Materials

The following are available online at https://www.mdpi.com/2073-4409/9/9/2119/s1, Figure S1: No differences in neuronal/axonal protein expression between C57 and DBA mice in whole brain extracts. Figure S2: Example trace of slow and fast wave conduction velocity in corpus callosum. Table S1: Sequences of primers used for PCR analysis. Supplemental Western blot data files: whole blot scans of Western blot data of Figure 4 and Figure S1.
