# Supplementary material for: Genetic Variation in CNS Myelination and Functional Brain Connectivity in Recombinant Inbred Mice
Source: Cells. 2020 Sep 18;9(9):2119. doi: 10.3390/cells9092119 (PMC7564997; doi:10.3390/cells9092119)

Supplemental
Western blot data

CNP

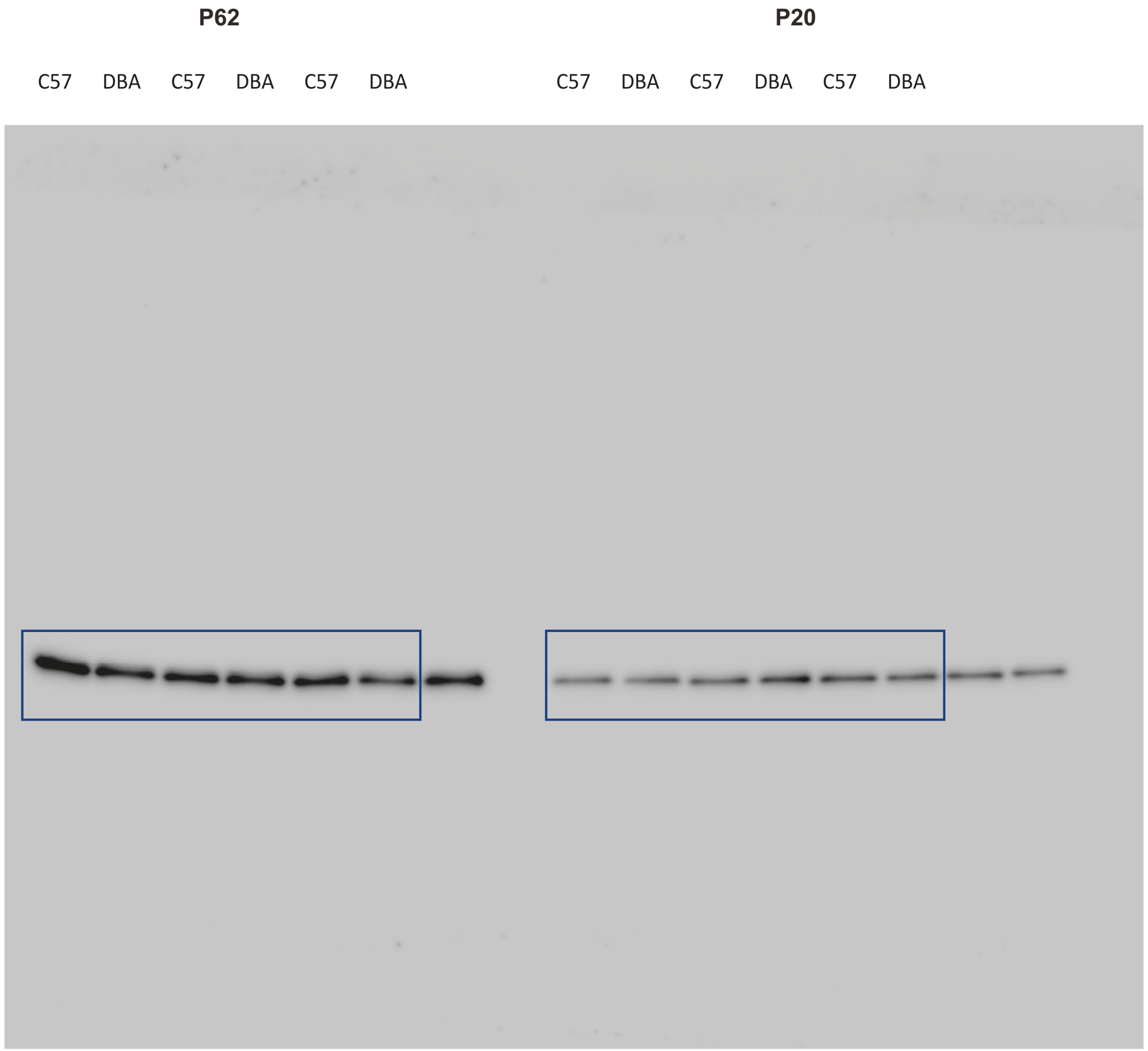

Supplemental
Western blot data

MOBP

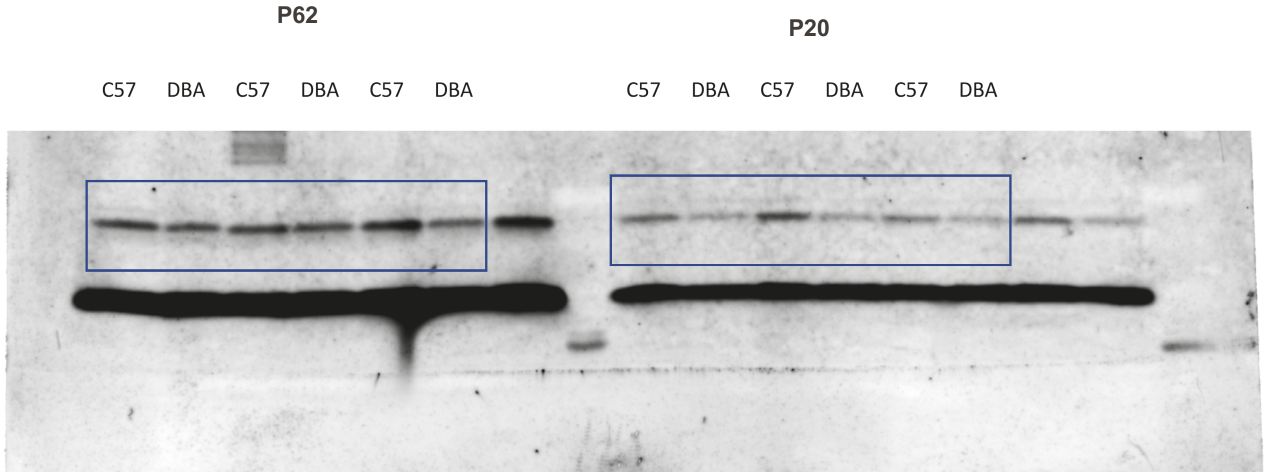

Supplemental
Western blot data

MAG

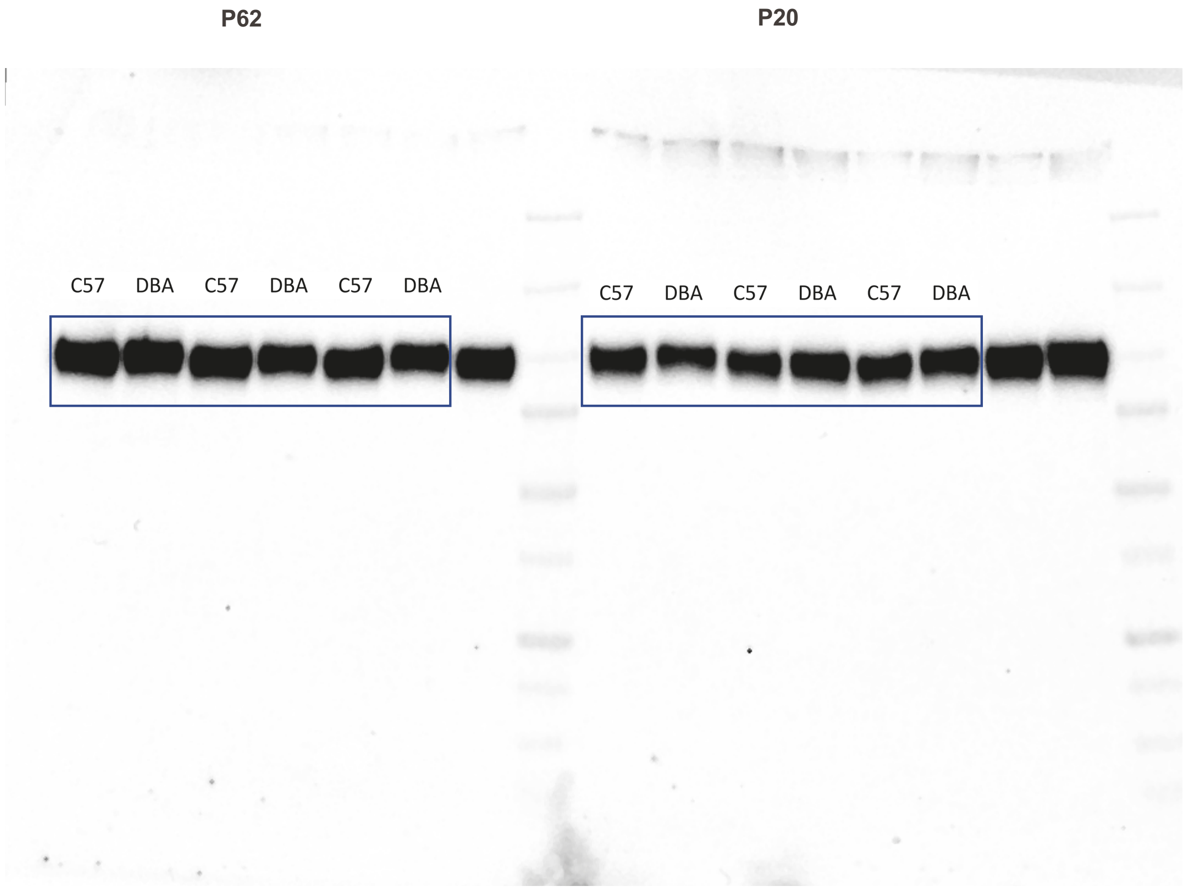

Supplemental
Western blot data

MOG

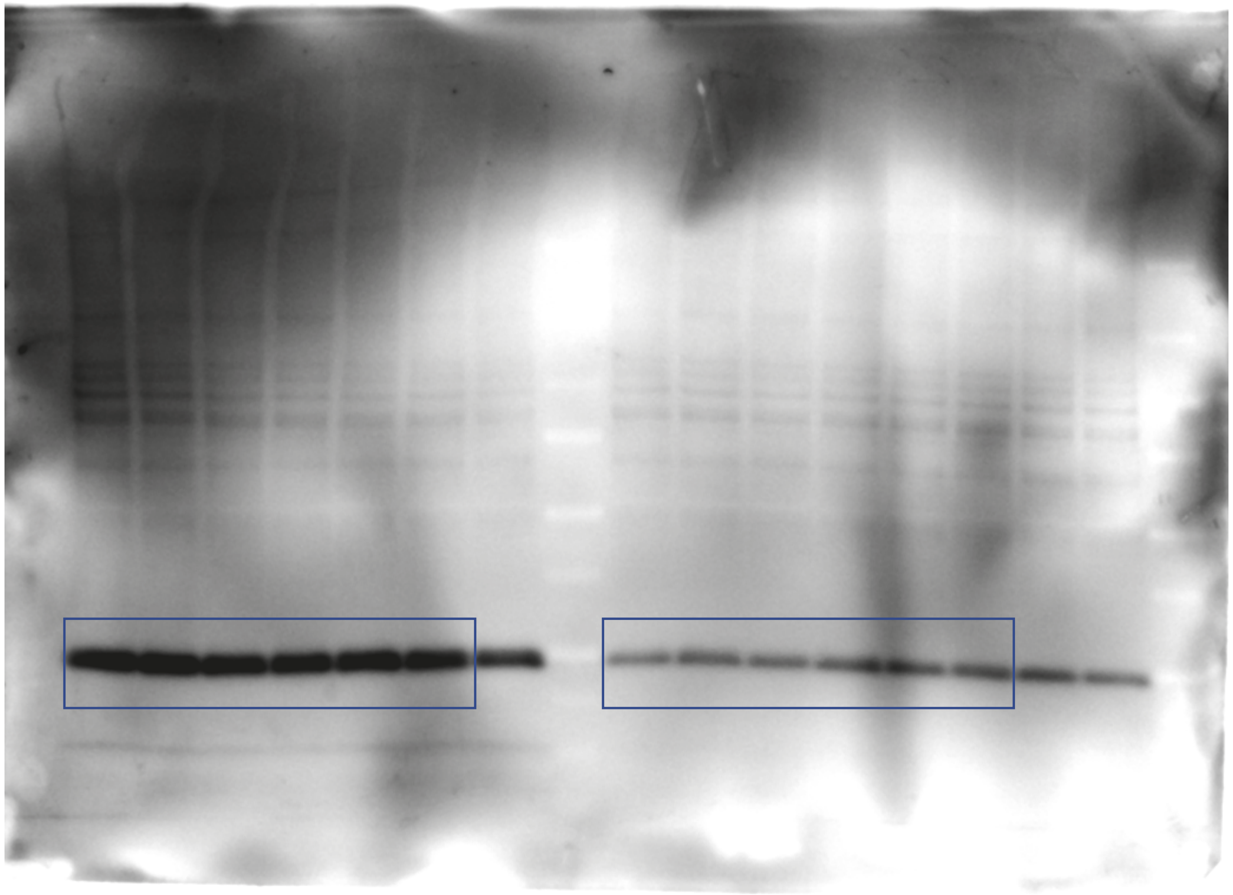

Supplemental
Western blot data

MBP

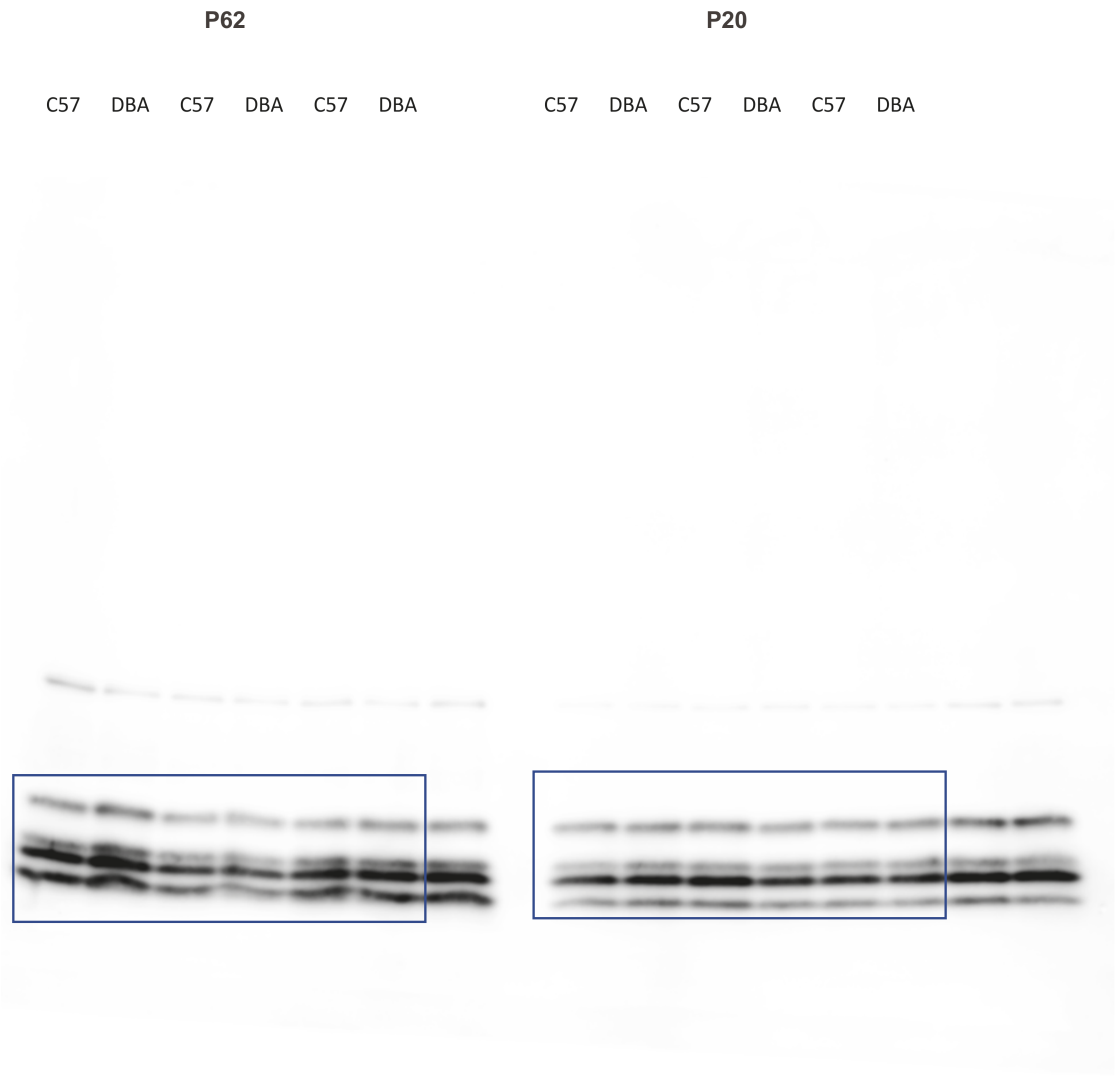

Supplemental
Western blot data

TUB3

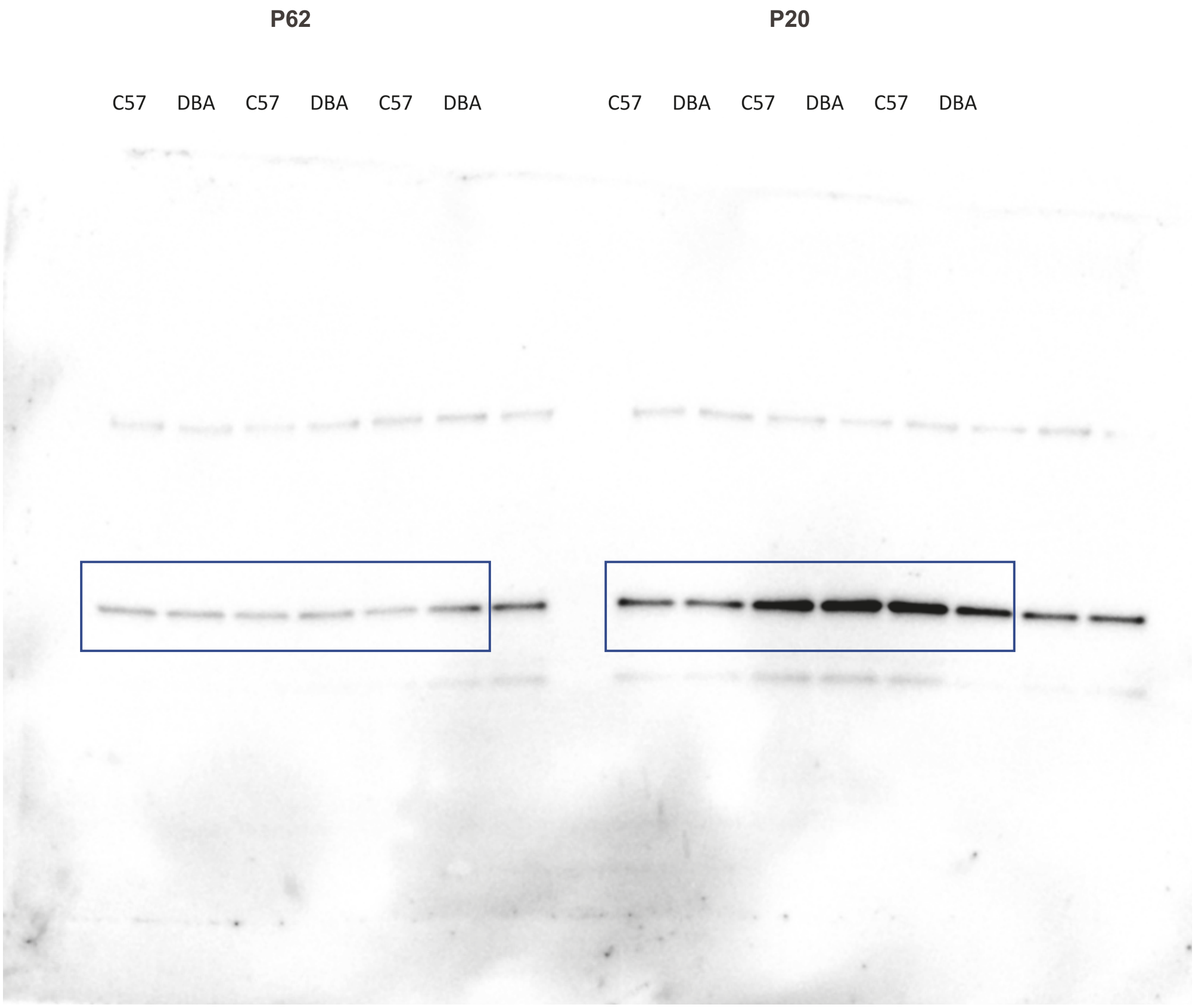

Supplemental
Western blot data

GAP43

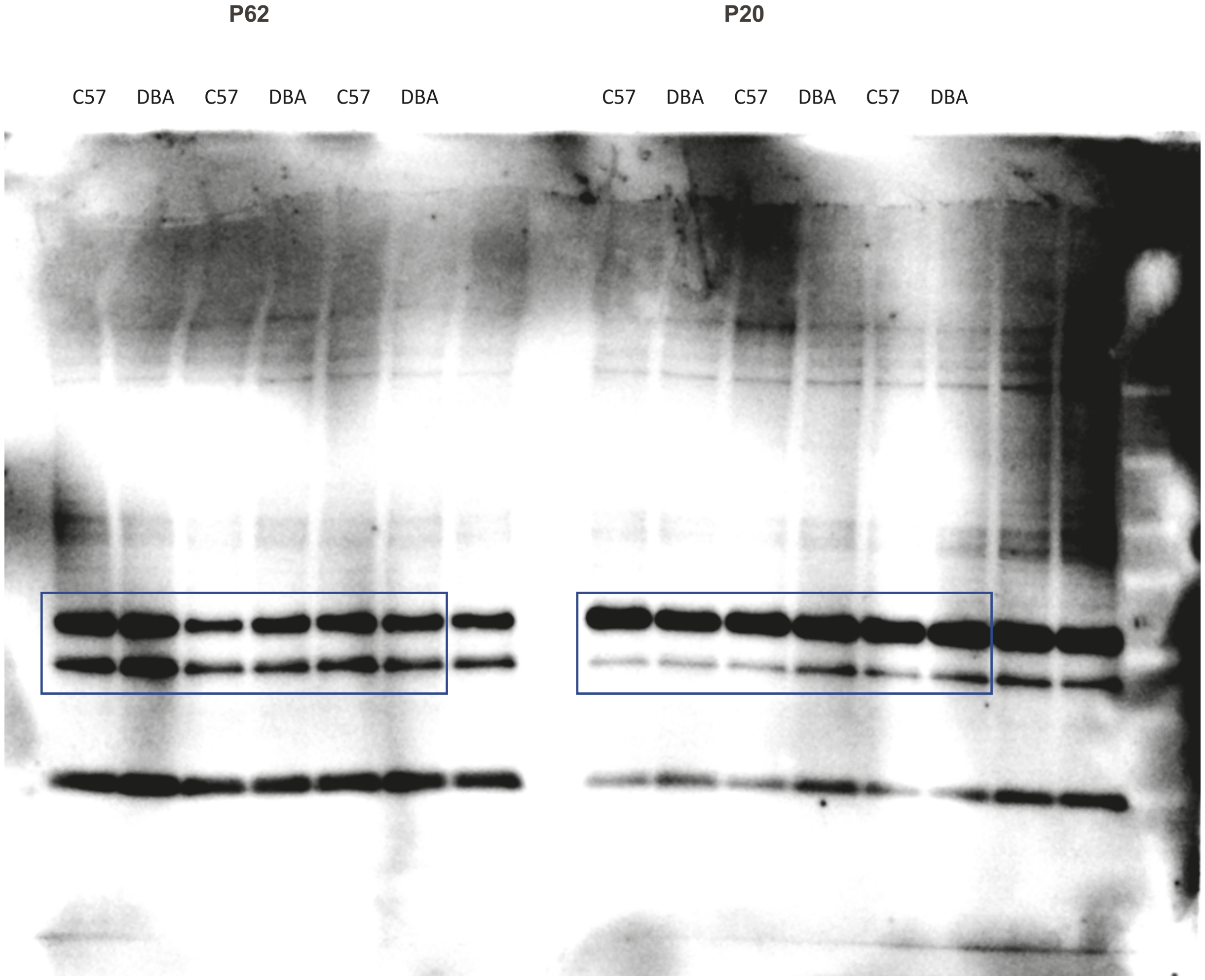

Supplemental
Western blot data

PSD95

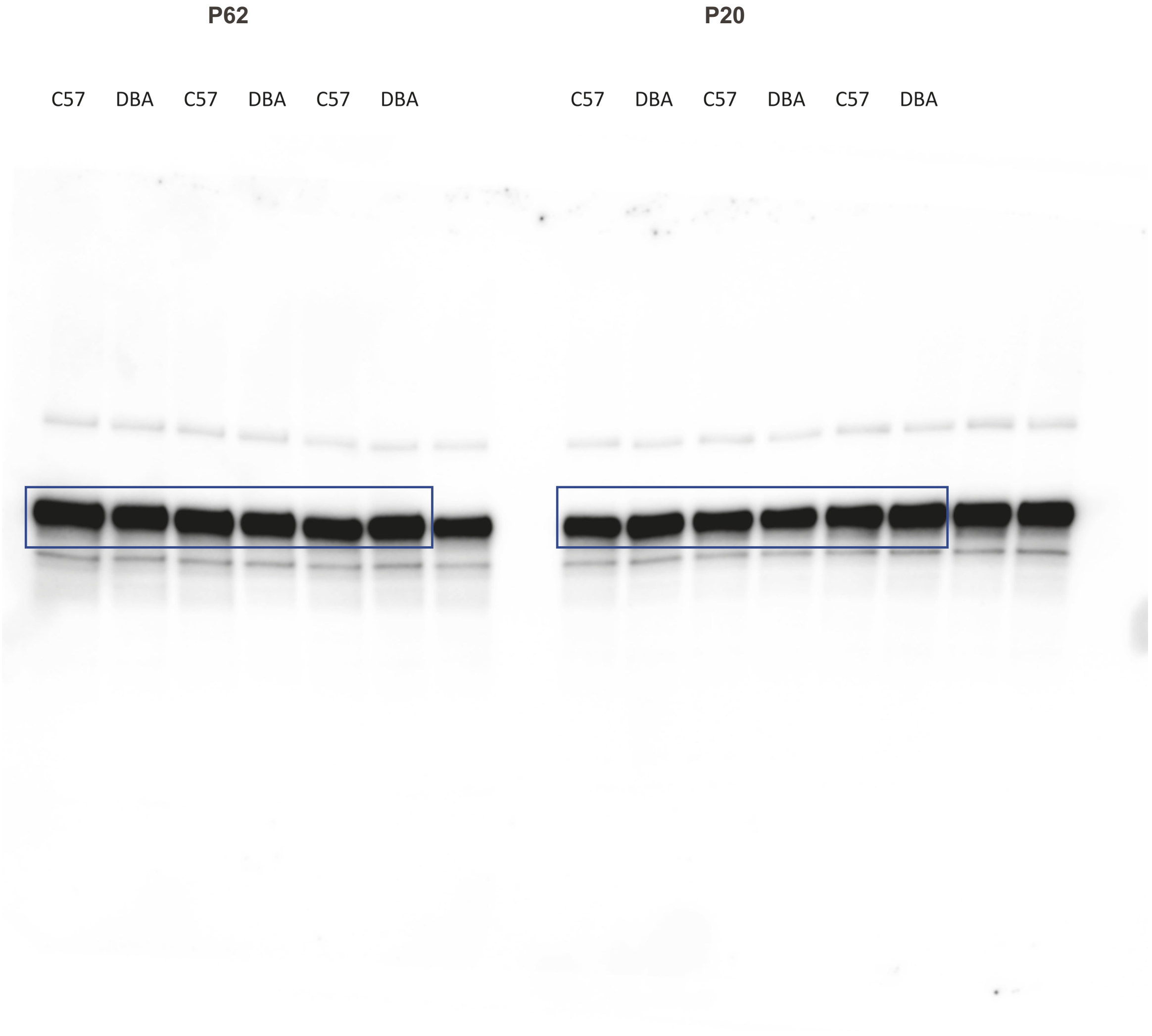

Supplement: Supplementary file 1 [file cells-09-02119-s001.zip › cells-930961-supplementary/original_images.v1.pdf]
