# Supplementary material for: Genetic Variation in CNS Myelination and Functional Brain Connectivity in Recombinant Inbred Mice
Source: Cells. 2020 Sep 18;9(9):2119. doi: 10.3390/cells9092119 (PMC7564997; doi:10.3390/cells9092119)
Supplement: Supplementary file 1 [file cells-09-02119-s001.zip › cells-930961-supplementary/supplementary.v3.pdf]

Figure S1: No differences in neuronal/axonal protein expression between C57 and DBA mice in whole brain extracts

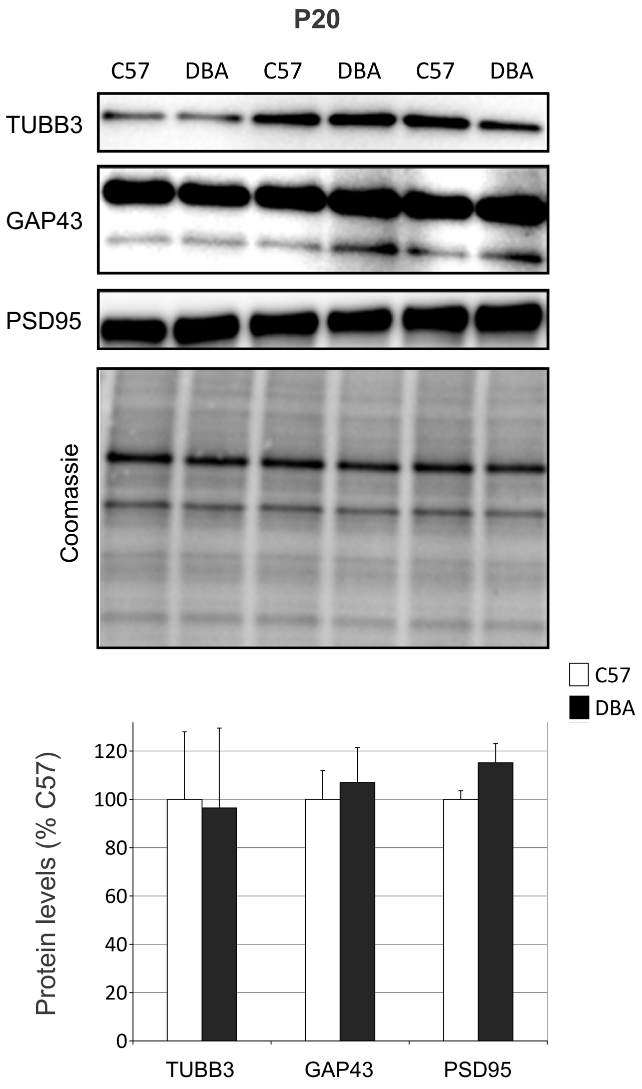

Figure S2: Example trace of slow and fast wave conduction velocity in corpus callosum.

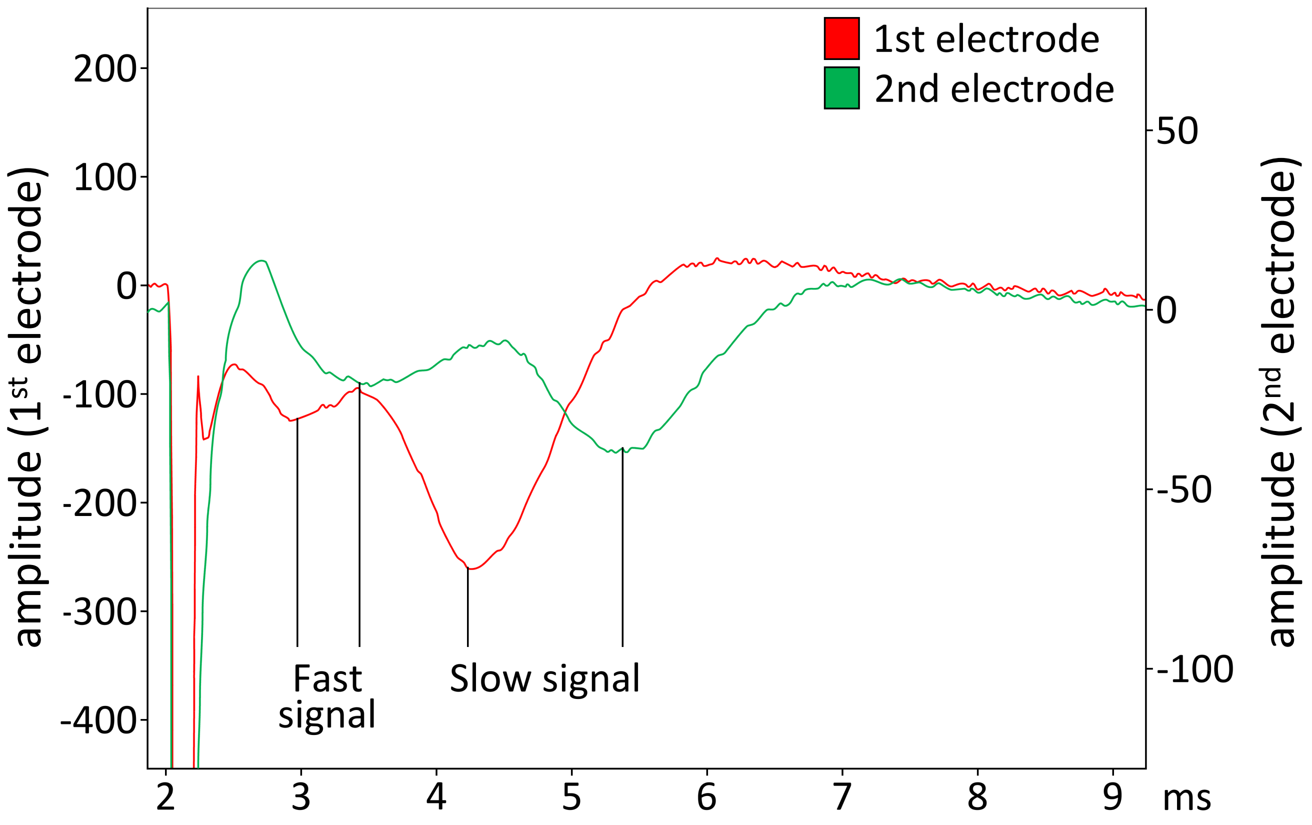

Supplemental Table S1

Table S1. Sequences of primers used for PCR analysis

Genes	Primer sequence forward	Primer sequence reverse
PLP1	CCCACCCCTATCCGCTAGTT	CAGGAAAAAAAGCACCATTGTG
MBP	CACACACGAGAACTACCCA	GGTGTTCGAGGTGTCACAA
MOBP	AACTCCAAGCGTGAGATCGT	CAGAGGCTGTCCATTCACAA
MAG	GATGATATTCCTCGCCACC	ACTGACCTCCACTTCCGTT
CNP1	AGGAGAAGCTTGAGCTGGTC	CGATCTCTTCACCACCTCCT
Claudin11	CACCATCCCCACCTGCCGAAAA	CAAGCCTGCACGTAGCCTGGAA
**Housekeeping genes**	**Primer sequence forward**	**Primer sequence reverse**
GAPDH	CAGCAATGCATCCTGCACC	TGGACTGTGGTCATGAGCCC
GDI	TTGCCAGCACCACTGTAGAGA	TCTGGTCAATGGGTTCCAACA
rEF1a	ACCCTCCACTTGGTCGTTTTG	AGCTCCTGCAGCCTTCTTGTC
Ghprt	ATGGGAGGCCATCACATTGT	ATGTAATCCAGCAGGTCAGCAA
VCP	TGGCCGTCTAGATCAGCTCAT	TTTCGCAGATTGGCTTTTAGG
